# Associations between gut microbiota and immune status in untreated B-cell lymphoma patients

**DOI:** 10.3389/fimmu.2025.1663066

**Published:** 2025-11-20

**Authors:** Jingxin Zhou, Jinrong Yao, Na Hu, Jing Su

**Affiliations:** 1 Department of Hematology, Suqian First People's Hospital Suqian, Suqian, Jiangsu, China; 2 Department of Hematology, The Second Affiliated Hospital of Fujian Medical University, Quanzhou, Fujian, China

**Keywords:** gut microbiota, B-cell lymphoma, 16s rDNA sequencing, pathological subtype, microecology-immune axis

## Abstract

**Background:**

Emerging evidence links gut microbiota to tumorigenesis via immune modulation, though subtype-specific microbial signatures in B-cell lymphomas remain unclear. This study explores microbiota-immune interactions across lymphoma subtypes to inform microbiota-targeted therapies.

**Methods:**

Twenty-seven treatment-naive B-cell lymphoma patients (8 DLBCL, 5 SLL, 5 FL, 7 MZL, 2 WM) and 20 HCs were enrolled. Fecal 16S rDNA sequencing, flow cytometry for immune cell subsets, and ELISA for cytokines/immunoglobulins were performed. Microbiota differences and correlations with immune parameters were analyzed.

**Results:**

B-cell lymphoma patients showed lower fecal microbiota richness/evenness (*P*<0.05), with increased Actinobacteriota, Bacilli, Enterobacteriaceae and decreased Bacteroidetes. Small B-cell lymphoma and DLBCL exhibited distinct flora: Selenomonadaceae/Actinobacteriota dominated in DLBCL, while Enterobacteriaceae prevailed in small B-cell subtypes. Correlations showed Enterobacteriaceae positively linked to Th cells/PCT/TNF and negatively to IL-10 in small B-cell lymphoma; Actinobacteriota correlated with B/T cells/Treg/IFN-β and inversely with IL-2/IL-4/CD8+T cells.

**Conclusions:**

This study identifies distinct patterns of gut microbiota dysbiosis across B-cell lymphoma subtypes and explores their correlations with host immune parameters.

## 1 Introduction

The human gut harbors over 100 trillion microorganisms, ten times the number of host somatic cells. This dynamic microbial organ plays a vital role in host physiology through symbiotic interactions, affecting processes such as nutrient metabolism and immune homeostasis (1). Extensive evidence shows that gut microbiota is involved not only in basic functions like nutrient processing and immune maturation but also in diseases such as diabetes, cardiovascular conditions, and immune-mediated intestinal disorders (2). In oncology, gut microbiota has dual tumor-modulating roles: it may promote carcinogenesis via pro-inflammatory metabolites and barrier disruption, or suppress tumors through immune activation and improved immunotherapy efficacy (3, 4). Microbial homeostasis is essential for treatment outcomes (5). Dysbiosis weakens the intestinal barrier and disrupts mucosal immunity, leading to chronic inflammation (6, 7), disrupting immune regulation (e.g., Th17/Treg balance), weakening tumor surveillance, and affecting drug metabolism (8).

B-cell lymphoma, which is a major non-Hodgkin lymphoma (NHL) subtype, comprises biologically diverse tumors originating from B lymphocytes (9). As the most typical aggressive subtype (~30% of NHL), diffuse large B-cell lymphoma (DLBCL) shows rapid lymph node growth and extranodal involvement (10). Other indolent subtypes, such as follicular lymphoma (FL), small lymphocytic lymphoma (SLL), and marginal zone B-cell lymphoma (MZL), progress slowly but can transform or relapse (11, 12). Despite treatment advances, many patients relapse or remain incurable, especially in indolent subtypes, posing long-term challenges (13, 14). Thus, identifying new therapeutic targets is essential.

Recent research has revealed distinctive gut microbiota alterations in DLBCL patients, including reduced diversity and increased pathogenic bacteria (e.g., Enterobacteriaceae). These microbial changes correlate with tumor burden and prognosis (15, 16). However, most studies focus solely on DLBCL, lacking comparative analyses across different aggressive B-cell lymphoma subtypes. A broader investigation of gut microbiota, disease features, and immune profiles across subtypes may aid in refining classification, prognostic tools, and early diagnosis.

This study recruited treatment-naive B-cell lymphoma patients (DLBCL, SLL, FL, MZL, Waldenström’s macroglobulinemia (WM)) and healthy controls (HCs), using 16S rDNA sequencing to profile gut microbiota. We compared microbial compositions between patients and controls and across subtypes and examined their associations with immune parameters (cell subsets, cytokines, immunoglobulins). Our aim is to clarify the microbiota–immune axis in B-cell lymphoma and support subtype-specific precision medicine through multi-subtype comparative analysis.

## 2 Methods and materials

### 2.1 Participant characteristics

Case enrollment: From January 2023 to December 2024, 27 consecutive treatment-naïve B-cell lymphoma patients were recruited at Suqian First People’s Hospital. The cohort included: Diffuse large B-cell lymphoma (DLBCL, n=8), Small lymphocytic lymphoma (SLL, n=5), Follicular lymphoma (FL, n=5), Marginal zone lymphoma (MZL, n=7), Waldenström macroglobulinemia (WM, n=2), Twenty age- and sex-matched HCs were enrolled. This study protocol complied with the Declaration of Helsinki and was endorsed by the Institutional Review Board of Suqian First People’s Hospital. All participants provided written informed consent.

Inclusion criteria:1) Pathologically confirmed B-cell lymphoma (per WHO 2022 classification) without prior anti-tumor therapy. 2) Age ≥20 years. 3) Signed informed consent.

Exclusion criteria:1) Comorbid inflammatory bowel disease, gastrointestinal surgery history, or enteral/parenteral nutrition dependence. 2) Acute infections, antibiotic use, immunosuppressants, or corticosteroids within 1 month. 3) Concurrent malignancies or autoimmune disorders.

Clinical Data Collection: Comprehensive clinicopathological parameters were systematically documented, including gender, age, histological subtype (classified per WHO 2022 criteria), disease stage (Ann Arbor staging system), International Prognostic Index (IPI), tumor burden biomarkers (serum lactate dehydrogenase (LDH) and β2-microglobulin levels), and extranodal involvement status. All pathological diagnoses underwent centralized review by two independent hematopathologists specializing in lymphoma. Cases were stratified into two biologically distinct cohorts based on disease aggressiveness: 1) aggressive DLBCL subgroup, and 2) indolent small B-cell lymphoma subgroup encompassing SLL, FL, MZL, and WM.

### 2.2 The collection of fecal and the analysis of 16S rDNA

The collection of fecal: Fresh fecal samples from patients at diagnosis and HCs were collected in aseptic preservation tubes containing anti-DNA degradation solution (CJ Bioscience Inc., Seoul, Korea) and stored at -80°C.

DNA extraction and sequencing: Stool sample DNA was extracted by using the QIA amp Fast DNA Stool Mini Kit (Qiagen, USA) according to the instruction from manufacturer. DNA purity and concentration were measured by NanoDrop 2000. Primers 341F (5’-CCTACGGGNGGCWGCAG-3’) and 806R (5’-GGACTACHVGGGTATCTAAT-3’) were used for 16S rRNA gene V3-V4 region amplification. A total of 25μL PCR reaction system was used, including 12.5μL 2×Taq Master Mix, 1μL each of primers F and R, and 10 ng of DNA template. These amplification conditions were configured as needed. PCR products were confirmed by 2% agarose gel electrophoresis and then sequenced with the Illumina NovaSeq platform (PE250 mode).

Data processing: Raw sequencing data were processed using FLASH assembly, Cut adapt primer trimming, and low-quality read filtering. Operational taxonomic units (OTUs) were clustered at a 97% similarity threshold and taxonomically annotated using the SILVA 138 database. Alpha diversity indices (Chao1, ACE, Shannon, Simpson) were calculated to estimated microbial richness and evenness. Beta diversity was evaluated via weighted/unweighted UniFrac distances and visualized using principal coordinate analysis (PCoA).

### 2.3 Statistical analysis of differences in microbial community composition and abundance

Alpha diversity metrics (Shannon, Simpson, ACE, Chao1) were computed using the R vegan package. Beta diversity was evaluated through unweighted and weighted UniFrac distances, with PCoA plots generated for visualization. All analysis were conducted in R v3.4.1. Linear discriminant analysis effect size (LEfSe) was proceeded on the Galaxy platform (https://galaxyproject.org).

### 2.4 Peripheral blood immune marker detection

5 mL of fasting peripheral venous blood was collected from the participants. Serum levels of IL-10, IL-6, IL-4, IL-2, IFN-γ, and TNF-α were quantified using enzyme-linked immunosorbent assay (ELISA, Beckman, USA). IgM, IgG, and IgA concentrations were measured with an IMMAGE-800 immunochemistry system (Beckman, CA, USA) and matched reagent kits.

### 2.5 Flow cytometry

2 ml of fasting peripheral venous blood was collected from the participants., and 100 μL aliquots were stained with fluorescently labeled antibodies: CD3-FITC, CD8-APC-Cy7, CD4-PC7, CD16-PE and CD19-APC (625642, BD Biosciences, CA, USA), CD3-FITC (570821, BD Biosciences, CA, USA), CD4-APC (561841, BD Biosciences, CA, USA), CD25-PECy7(571741, BD Biosciences, CA, USA), CD127-PE(571814, BD Biosciences, CA, USA). After dark incubation and erythrocyte lysis, 10, 000 cellular events were collected by using a FACSCanto™ flow cytometer (BD, USA). Data was analyzed with FlowJo software.

### 2.6 Statistical methods

Alpha-diversity was estimated using Shannon, Simpson, ACE, and Chao1 indices with the R vegan package. Beta-diversity was assessed by calculating weighted and unweighted UniFrac distance matrices and visualized using Principal Coordinate Analysis (PCoA) plots. Group differences in β-diversity were statistically evaluated using PERMANOVA via the adonis2 function in the R vegan package, with 999 permutations to determine significance. Differentially abundant taxa were identified using Linear Discriminant Analysis Effect Size (LEfSe) on the Galaxy platform (www.huttenhower.sph.harvard.edu/galaxy/), with a linear discriminant analysis (LDA) score ≥2.0 and P<0.05 considered significant. Associations between microbial abundance and immune parameters were assessed using Spearman correlation.

Statistical analyses for clinical data were performed using SPSS 23.0. Measurement data are represented as M (P25, P75), and comparisons between multiple groups were made using the Kruskal-Wallis H test, with pairwise comparisons between groups using the Mann-Whitney U test. Bonferroni *post hoc* tests were analyszed to confirm the statistical significance. Categorical variables were expressed as frequencies and percentages and compared using the Fisher’s exact test or Chi-square test, as appropriate. Continuous variables were tested for normality; normally distributed data were expressed as mean±standard deviation (SD) and compared using independent sample t-tests, whereas non-normally distributed data were expressed as median and interquartile range (IQR). R v3.4.1 was used for data visualization, and a two-sided P<0.05 was considered statistically significant.

## 3 Results

### 3.1 Baseline characteristics of the study population

A total of 27 untreated B-cell lymphoma patients and 20 HCs were included in this study. In the B-cell lymphoma group, there were 8 cases of DLBCL, 5 cases of SLL, 5 cases of FL, 7 cases of MZL, and 2 cases of WM. Among the patients, 16 (59.3%) were male, and the median age was 60 years. Six patients (22.2%) were diagnosed at stage I–II and 21 (74.1%) at stage III–IV. Extranodal involvement was present in 17 cases (63.0%), with gastrointestinal involvement in 2 cases (7.4%). Elevated lactate dehydrogenase (LDH≥250 U/L) was observed in 7 patients (25.9%), and β2-microglobulin levels≥3 mg/L were seen in 12 patients (44.4%) (**Table 1**).

**Table 1:** Baseline characteristics of untreated B-cell lymphoma patients and healthy controls: demographic, clinical, and immunophenotypic features.

Variables	Total	DLBCL group	Small B group	P value
Number of patients	27	8	19	–
Gender (male%)	16(59.3)	5(62.5)	11(57.9)	0.824
Age (years)	60.4±13.9	62.5±21.8	59.5±9.5	0.621
WBC (10*9/L)	12.5±12.9	5.9±1.9	15.1±14.6	0.013
ANC	4.3±3.0	5.1±4.3	3.9±2.3	0.354
LYM	1.5(1.0, 2.6)	1.3(0.5, 1.5)	1.9(1.1, 16.9)	0.030
RBC	4.0±0.7	4.2±0.5	3.9±0.8	0.419
Hb (g/L)	119.0±21.8	126.3±14.7	116.0±23.8	0.272
PLT (10*9/L)	161.0(94.0, 208.0)	204.0(145.7, 249.3)	143.0(86.0, 195.0)	0.075
RET	2.4±1.7	2.2±0.9	2.4±2.7	0.896
UA (mmol/L)	132.9±71.8	146.6±148.6	127.1±116.2	0.716
D-dimer(mg/L)	220.5±228.0	338.7±254.9	162.2±206.2	0.080
LDH (U/L)	214.8±89.8	286.3±92.7	188.5±74.9	0.011
Ferritin (μg/L)	189.6±210.7	230.1±165.8	171.6±229.8	0.524
β2-microglobulin(mg/L)	3.8±4.2	2.7±0.8	4.3±4.9	0.363
TB	27.9±24.8	22.6±19.4	30.1±26.9	0.480
ALB	37.8±4.9	37.0±3.2	38.2±5.5	0.581
ALT	17.1±6.5	19.0±8.7	16.3±5.4	0.337
AST	19.3±7.4	21.8±11.7	18.2±4.7	0.271
GGT	26.1±16.6	37.1±21.8	21.4±11.6	0.087
CREA	270±196.8	275.5±154.5	267.7±215.9	0.927
IL-10 (pg/mL)	7.6(4.9, 17.8)	10.1(4.9, 18.4)	6.1(4.6, 17.8)	0.567
IL-6 (pg/mL)	12.3(5.5, 35.5)	7.6(4.6, 35.8)	14.8(8.8, 35.5)	0.285
IL-2 (pg/mL)	1.5±0.8	1.5±0.6	1.5±0.9	0.877
IL-4 (pg/mL)	1.2±0.9	1.2±0.8	1.8±1.0	0.873
TNF	2.5(2.0, 4.5)	2.3(1.5, 4.8)	2.6(2.1, 5.6)	0.350
IFN-B	2.2(1.8, 2.6)	2.4(1.6, 3.4)	2.0(1.8, 2.4)	0.660
lgM (g/L)	0.8(0.5, 2.5)	0.8(0.5, 2.2)	0.9(0.5, 74.9)	0.657
lgA (g/L)	1.8(0.9, 8.0)	2.2(1.8, 3.1)	1.2(0.6, 46.4)	0.458
C3	0.9(0.8, 1.5)	1.0(0.8, 1.2)	0.9(0.8, 62.3)	0.534
C4	0.2(0.2, 0.4)	0.2(0.2, 0.3)	0.2(0.2, 6.2)	0.790
APTT	1.0(1.0, 25.0)	1.0(0.9, 20.7)	1.0(1.0, 25.0)	0.696
TT	16.0(14.3, 17.5)	13.6(13.4, 16.5)	16.6(15.4, 17.6)	0.008
FIB	3.1(2.5, 4.0)	4.1(3.5, 5.5)	2.8(2.4, 3.9)	0.051
CD3+Total Lymphocyte Count(/ul)	2284.3±5231.5	882.6±393.3	2874.5±6183.1	0.377
CD4+Tcell count(/ul)	714.9±528.2	502.3±257.1	821.2±603.5	0.446
CD8+Tcell count(/ul)	543.8±576.3	240.5±129.7	695.5±656.0	0.296
Treg	8.2±2.7	7.1±1.8	8.7±2.9	0.243
Stages (%)				
I+II	7(29.6)	3(37.5)	4 (21.1)	0.332
III+IV	20(74.1)	5(62.5)	15 (73.9)	
low risk	6 (22.2)	1 (12.5)	5 (26.3)	-
intermediate risk	8 (29.6)	2 (25.0)	6 (31.6)	-0.293
high risk	13 (48.1)	5 (62.5)	8 (42.1)	-
Extranodal disease	17 (63.0)	4 (50)	13 (68.4)	0.316-
Serum β2-microglobulin >3 mg/L	12 (44.4)	3 (37.5)	9 (47.4)	0.484-
Bone marrow involvement	2 (7.4)	0	2 (10.5)	0.487
Involvement of the gastrointestinal tract	2 (7.4)	1 (12.5)	1 (5.3)	0.548
Splenomegaly	9 (33.3)	2 (25.0)	7 (36.8)	-0.450
Ki-67≥50%	12 (44.4)	7 (87.5)	5 (26.3)	-0.006

In the DLBCL group, 12.5% of participants were classified as low-risk, 25.0% as intermediate-risk, and 62.5% as high-risk. None of the DLBCL patients had bone marrow involvement, while 12.5% had gastrointestinal tract involvement, 25.0% had splenomegaly, and 87.5% showed Ki-67≥50%. In contrast, among patients with small B-cell lymphoma (including SLL, FL, MZL, and WM), 26.3% were low-risk, 31.6% intermediate-risk, and 42.1% high-risk. Bone marrow involvement was observed in 10.5%, gastrointestinal involvement in 5.3%, splenomegaly in 36.8%, and Ki-67≥50% expression in 26.3% of cases (**Table 1**).

### 3.2 Gut microbiota alterations in treatment-naive B-cell lymphoma patients

**Figure 1:**
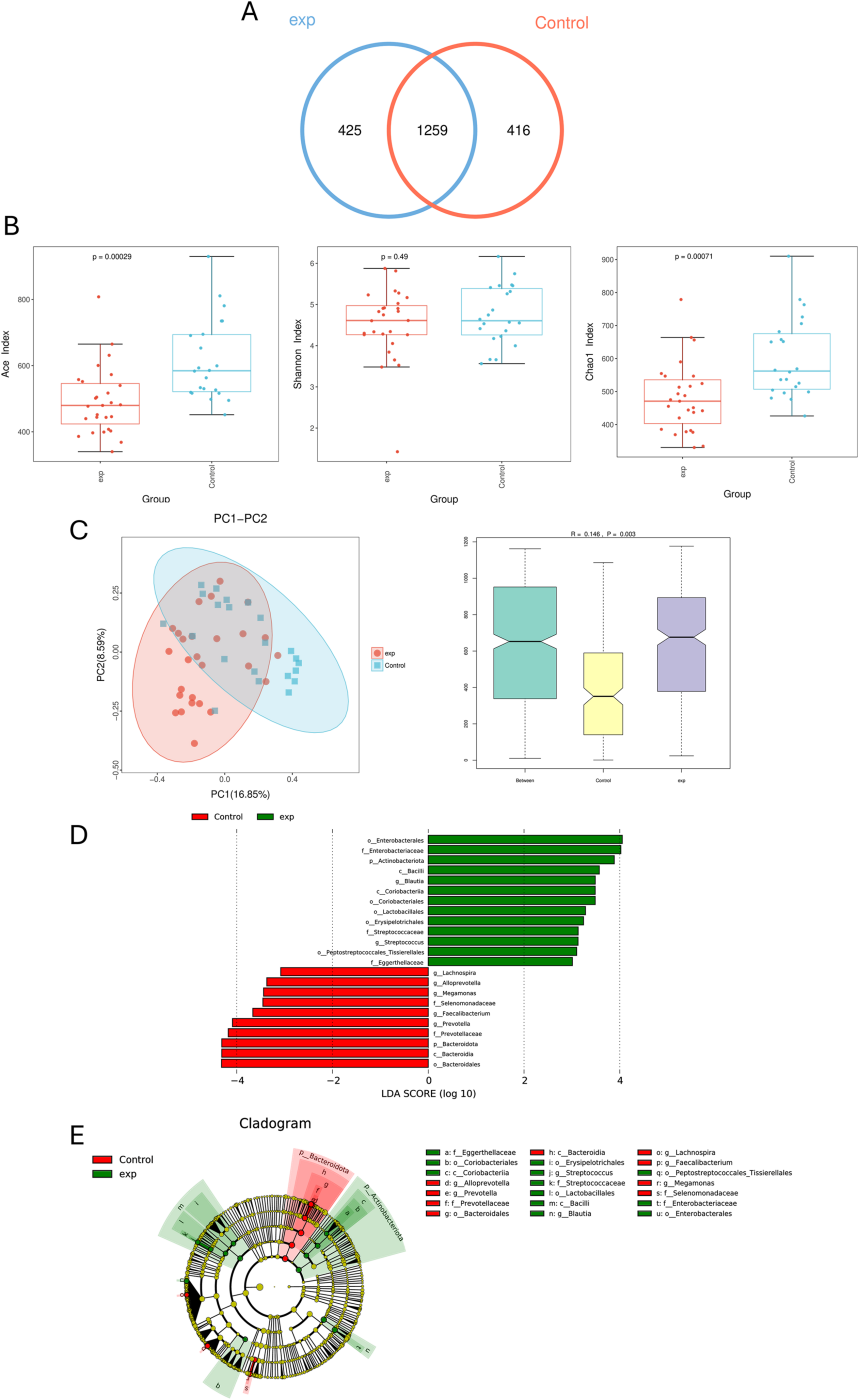
Comparing the gut microbiota of untreated patients with B-cell lymphoma (exp) and healthy control (HCs). **(A)** Similarity analysis between otus of gut microbiota from patients and HCs in B-cell lymphoma patients. **(B)** Alpha diversity analysis of B-cell lymphoma patients and HCs. **(C)** Beta diversity analysis of B-cell lymphoma patients and HCs. **(D)** LDA shows species with significant differences in abundance between B-cell lymphoma patients and HCs. **(E)** Evolutionary diagram: circles arranged radially from inside to outside indicate taxonomic levels from phylum to genus. Green (exp group) nodes indicate more abundant classification types in the B-cell lymphoma group, and Red (control group) nodes indicate more abundant classification types in HCs.

Analysis of 47 fecal samples through 16S rDNA gene sequencing yielded 3, 747, 313 high-quality sequences, with an average of 76, 475 sequences in every sample. OTU analysis revealed 425 unique OTUs in the B-cell lymphoma group and 416 unique OTUs in HCs, with 1, 259 shared OTUs between groups (**Figure 1A**).

Alpha diversity indices reflect diversity (Shannon) and microbial richness (ACE and Chao1) demonstrated significantly higher ACE (*P*<0.05) and Chao1 (*P*<0.05) indices in HCs compared to lymphoma patients. Although no statistically significant difference in Shannon index was observed between groups (*P* > 0.05), HCs exhibited a higher trend compared to lymphoma patients, suggesting greater microbial richness, evenness, and ecosystem stability in controls (**Figure 1B**). β-diversity analysis showed that the composition of intestinal microbial community in HCs group was significantly different from that in B-cell lymphoma group (*P* = 0.003) (**Figure 1C**).

LEfSe analysis identified differentially abundant taxa between groups. The lymphoma group showed enriched relative abundance of p_Actinobacteriota, c_Bacilli, o_Enterobacteriales, f_Enterobacteriaceae, and g_Bacilli. Conversely, O_Bacteroidetes demonstrated higher abundance in HCs (**Figures 1D, E**).

### 3.3 Differences in immune parameters among untreated patients with different B-cell lymphoma subtypes

27 cases of B-cell lymphoma participated in this study were divided into two groups according to the degree of disease invasion, one group was DLBCL, and another group was small B-cell lymphoma. The differences of immune indexes between the two groups were statistically analyzed. There were 5 male and 3 female participants with a mean age of 62.5 in the DLBCL group, and 11 male and 8 female participants with a mean age of 59.5 in the small B-cell lymphoma group. There were no statistically significant differences in age and gender between two groups. The differences of blood routine and basic biochemical indexes between the two groups were analyzed by venous blood samples. The WBC and LYM levels in DLBCL group were significantly lower than the small B-cell lymphoma group (5.9±1.9 vs. 15.1±14.6×10^9^/L, and median 1.3 vs. 1.9×10^9^/L; *P* = 0.013, 0.030). LDH (U/L) in DLBCL group was 286.3±92.7, which was significantly higher than the Small B group 188.5±74.9 (*P* = 0.011). In terms of coagulation function, the TT value of DLBCL group was significantly lower than the Small B group (13.6 vs. 16.6, *P* = 0.008).

### 3.4 Gut microbiota alterations in treatment-naive patients with different B-cell lymphoma subtypes

**Figure 2:**
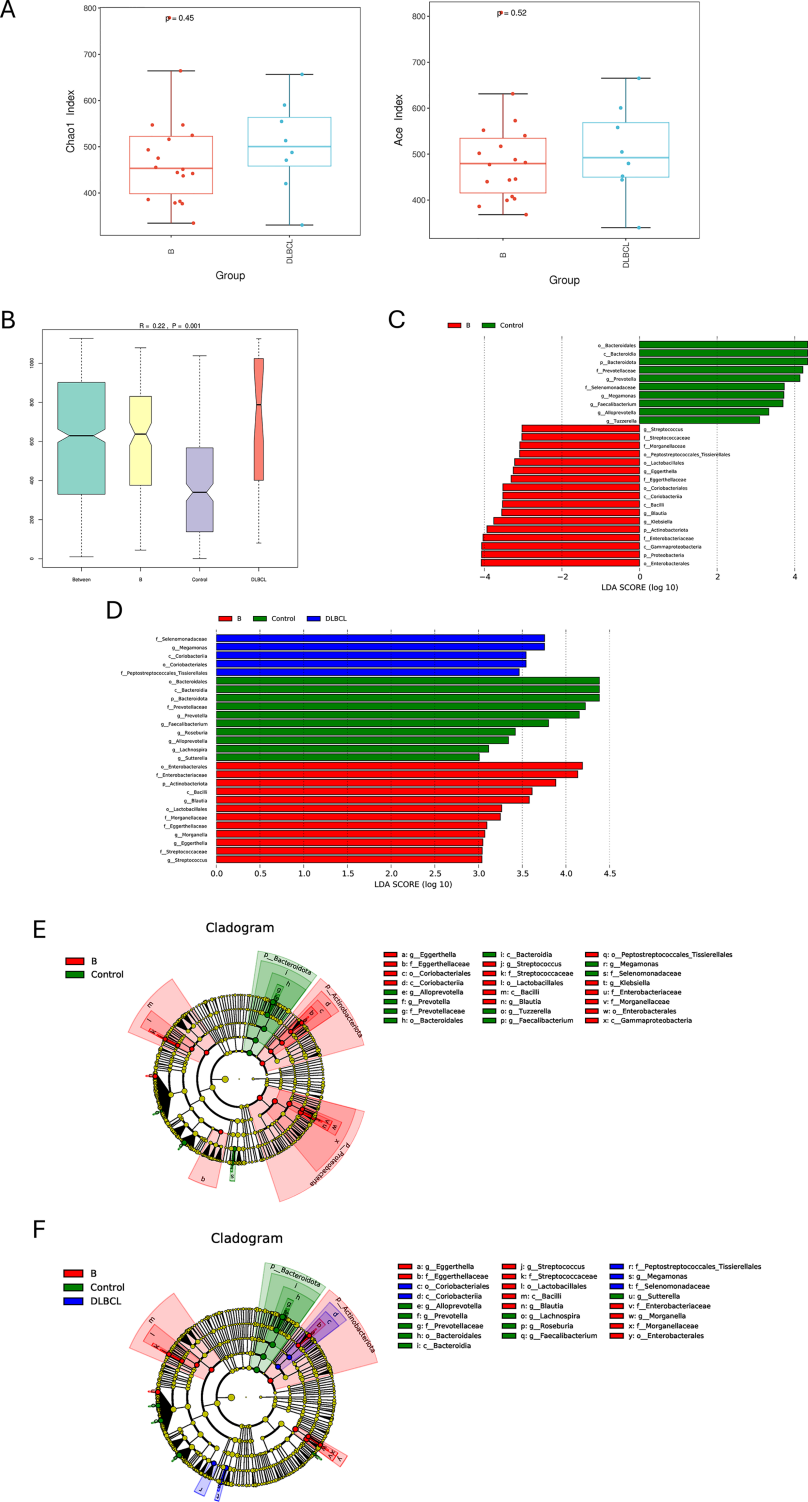
Comparison of gut microbiota among patients with DLBCL, small B-cell lymphoma, and healthy controls. **(A)** α-diversity analysis of DLBCL and small B-cell lymphoma patients. **(B)** β-diversity analysis of DLBCL and small B-cell lymphoma patients. Between denotes differences between groups, B denotes small B-cell lymphoma group, Control denotes healthy controls, and DLBCL denotes DLBCL group. **(C)** LDA shows species with significant differences in abundance between small B-cell lymphoma patients and HCs. **(D)** Evolutionary diagram: circles arranged radially from inside to outside indicate taxonomic level from phylum to genus. Red nodes indicate more abundant classification types in the small B-cell lymphoma group, and green nodes indicate more abundant classification types in HCs. **(E)** LDA revealed species with significant differences in abundance between DLBCL and small B-cell lymphoma patients. **(F)** Evolutionary diagram: circles arranged radially from inside to outside indicate taxonomic level from phylum to genus. Red nodes indicate more abundant classification types in the small B-cell lymphoma group, and blue nodes indicate more abundant classification types in DLBCL.

Differences in gut microbiota between the two groups and the difference in gut microbiota between the two groups and HCs group were analyzed. The alpha diversity index results showed that the ACE and Chao1 index in the small B-cell lymphoma group tended to be lower than the DLBCL group, but results were not significant (*P* > 0.05) (**Figure 2A**). The results of beta-diversity analysis showed that gut microbial community composition was significantly different between the small B-cell lymphoma, DLBCL, and HCs groups (*P* = 0.001) (**Figure 2B**).

LEfSe analysis found that in the small B-cell lymphoma group, the relative abundance of taxa was higher, including p_Actinobacteriota, c_Bacilli, f_Enterobacteriaceae, o_Enterobacteriales and g_Blautia. The relative abundance of P_Bacteroidetes, O_Bacteroidetes and c_Bacteroidetes was higher in HCs group (**Figures 2C, D**). Compared with the HCs group, the dominant bacteria in the DLBCL group were p_selenomonadaceae and c_Actinobacteriota, while the dominant bacteria in the small B-cell lymphoma group were f_Enterobacteriaceae (**Figures 2E, F**). These results indicate that although both small B-cell lymphoma and DLBCL are derived from B-cell lymphoma, there are significant differences in the distribution of flora between small B-cell lymphoma and DLBCL, indicating that different flora are participated in the pathogenesis of different subtypes of B-cell lymphoma.

### 3.5 Correlation analysis between gut microbiota and host immune status

**Figure 3:**
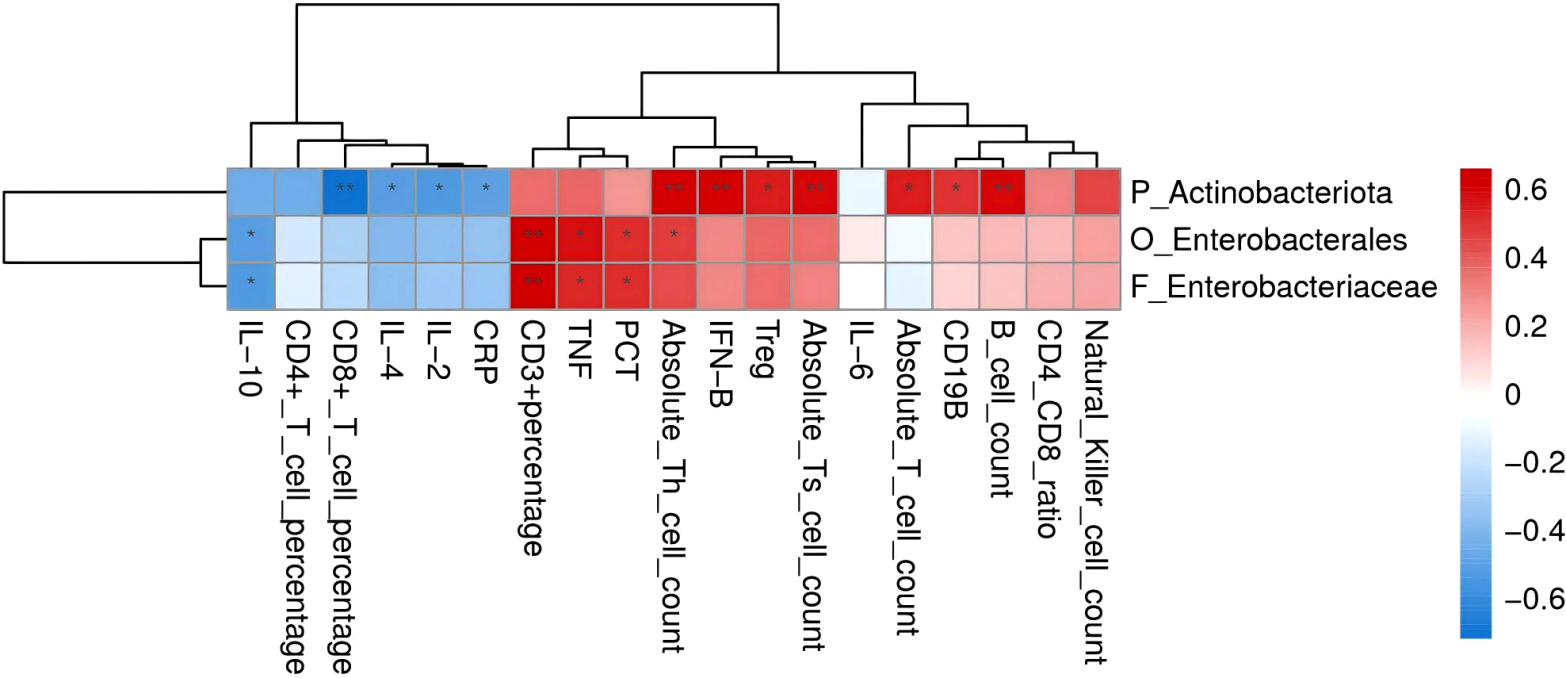
Heat map showing Spearman correlation between differential gut microbiota and immune indicators in the small B-cell lymphoma group. Red indicates positive correlation and blue indicates negative correlation. The darker the color, the greater the correlation coefficient.

To elucidate the potential interplay between subtype-specific gut microbiota and host immunity, we performed a Spearman correlation analysis between the differentially abundant flora (f_Enterobacteriaceae and p_Actinobacteriota) and a panel of immune parameters in the small B-cell lymphoma cohort (**Figure 3**). Notably, f_Enterobacteriaceae exhibited a significant positive correlation with TNF, PCT and CD3+T cell ratio, while showing a negative correlation with IL-10. Conversely, p_Actinobacteriota demonstrated strong positive correlations with multiple B-cell and T-cell subsets (B cell count, CD19+B cells, absolute T cell count, Tregs, and Th cells), as well as with IFN-B. It was negatively correlated with cytotoxic CD8+T cells and the cytokines IL-2 and IL-4 (**Supplementary Data 1, 2**). These findings imply that Actinobacteriota may influence lymphoma immunobiology by modulating regulatory and helper T cell balances.

## 4 Discussion

By systematically analyzing the characteristics of gut microbiota in participants with untreated B-cell lymphoma, this study revealed the composition and function of gut microbiota between different pathological subtypes (DLBCL and small B-cell lymphoma) and their dynamic association with the host immune microenvironment in a multi-subtype framework for the first time. Studies have not only confirmed the prevalence of intestinal flora imbalance in participants with B-cell lymphoma but also clarified the existence of subtype-specific flora markers, which provides a new microecological perspective for understanding the heterogeneity of lymphoma.

Compared with HCs group, B-cell lymphoma patients showed significantly lower ACE and Chao1 index of intestinal flora (*P*<0.05). The abundance of p_Actinobacteriota, c_Bacilli, and f_Enterobacteriales increased, while O_Bacteroidetes decreased. The two patients with gastrointestinal involvement showed no significant microbiota differences compared to non-involved cases, indicating no impact on the overall dysregulation pattern. This result indicates that the intestinal flora of participants with B-cell lymphoma shows a dual imbalance pattern of proliferation of pro-inflammatory bacteria and depletion of anti-inflammatory bacteria. The enrichment of p_Actinobacteriota was associated with inflammatory state. Wang et al. found that the proportion of p_Actinobacteriota increased after induction of acute pancreatitis in mice (17). The increased abundance of p_Actinobacteriota in DLBCL may contribute to the proinflammatory environment associated with the disease. This view is supported by Li’s study, which found that Actinobacteriota members are significantly increased in a mouse model of acute intestinal inflammation, and their reduction is a key feature of successful anti-inflammatory treatment (18). Therefore, we hypothesized that the increased abundance of actinobacteria in DLBCL may also be involved in the pathogenesis of DLBCL. Corynebacterium of this phylum is known to release cytokines IL-1β (19). This chronic inflammatory microenvironment, potentially driven by gut microbiota alterations present at diagnosis, may in turn promote abnormal activation and survival of B cells, thereby contributing to lymphomagenesis. Further functional experiments are needed to validate the causal role of these microbial changes in DLBCL pathogenesis.

The significant enrichment of O_Bacteroidetes in the HCs group suggests a potential protective effect against tumors in healthy people (20). Bacteroides (e.g., Bacteroides fragilis) are major producers of intestinal butyrate. As shao et al. found, Bacteroides fragilis promotes the secretion of short-chain fatty acids (SCFA) in the gut. It regulates the NLRP3-mediated inflammatory signaling pathway negatively, inhibits the secretion of proinflammatory mediators and activation of macrophages, reduces the intestinal inflammation level and limits the development of colitis-associated cancers (21). Furthermore, butyrate can upregulate the expression of tumor suppressor genes by inhibiting histone deacetylase (HDAC), and promote the differentiation of Treg cells by activating GPR109A receptor to maintain immune tolerance (22, 23). The decrease of Bacteroidetes in participants with B-cell lymphoma will lead to the decrease of butyrate synthesis, thereby weakening the intestinal barrier function and anti-tumor immune surveillance (24). Notably, although the abundance of Bacteroides in participants with small B-cell lymphoma was higher than that in the DLBCL group, it was still lower than that in healthy controls, suggesting that inflammatory metabolic disorder is a common feature of B-cell lymphoma, but its degree may vary depending on subtype aggressiveness.

There were significant microbiota types between DLBCL and small B-cell lymphoma. p_Selenomonadaceae and c_Actinobacteriota were the dominant flora in DLBCL patients. p_Selenomonadaceae is a family of anaerobic or microaerobic Gram-negative bacteria. The typical representatives of P_selenomonadaceae, such as Anaerococcus, are commonly colonized in the oral cavity and upper respiratory tract of healthy people (25). The enrichment of this flora in DLBCL reflects the impairment of intestinal barrier function and microbial translocation in these patients. The integrity of the intestinal barrier in patients with DLBCL may be compromised by tumor-associated inflammation or dysbiosis of the gut microbiota, leading to increased intestinal permeability (26). This barrier disruption allows the commensal bacteria Selenomonadaceae of the oral or upper respiratory tract to transgress to the gut through swallowing or blood circulation, forming abnormal colonization (27). In addition, after intestinal barrier breakdown, translocated bacteria or their products enter the circulation system, which may activate systemic inflammatory responses and promote lymphoma cell proliferation and immune escape. Selenomonadaceae release lipopolysaccharide (LPS) and other pathogen associated molecular patterns (PAMPs), activate TLR4/NF-κB pathway, enhance the secretion of pro-inflammatory factors, and cooperate with other pathogenic bacteria to further destroy intestinal homeostasis (27). Propionibacterium, the representative genus of c_Actinobacteriota, can metabolize dietary fiber to generate short-chain fatty acids (SCFAs), especially propionic acid (28). Propionic acid promotes the differentiation of Th17 cells and secrete pro-inflammatory factors by activating GPR41 receptor. There is an antagonistic relationship between the differentiation of Treg and Th17 cells. The excessive activation of Th17 cells can inhibit the immunosuppressive function of Treg cells, leading to immune imbalance. Therefore, in DLBCL, there is a high Th17/Treg ratio, which is related to disease development, drug resistance and poor prognosis.

The significant enrichment of f_Enterobacteriaceae in the intestinal flora of participants with small B-cell lymphoma is a typical marker of intestinal flora imbalance (27). Firstly, Enterobacteriaceae activate the TLR4/NF-κB pathway through LPS to induce the release of pro-inflammatory factors such as IL-6 and TNF-α, forming a chronic inflammatory microenvironment. In this study, f_Enterobacteriaceae was positively correlated with TNF levels, further supporting its proinflammatory role. This persistent inflammation may promote clonal proliferation of malignant B cells by activating of the B-cell receptor (BCR) signaling pathway (27). Second, genotoxins secreted by some strains of the Enterobacteriaceae, such as colibactin-producing Escherichia coli, can induce DNA double-strand breaks in the host, leading to genomic instability and promoting oncogene mutations or epigenetic abnormalities, thereby accelerating the transformation of indolent lymphoma to aggressive disease (28, 29). In addition, the negative correlation between Enterobacteriaceae and anti-inflammatory factor IL-10 (*P*<0.05) suggested that Enterobacteriaceae might break the balance of Th1/Th17 and Treg by inhibiting immune tolerance, and further aggravate the stimulation of malignant cells by inflammation (30).

The difference in the characteristics of flora between DLBCL and small B-cell lymphoma provides a new idea for precision diagnosis and treatment. The characteristics of dominant Actinobacteria and the imbalance of Th17/Treg are related to the aggressive phenotype of DLBCL. Its abundance can be used as an indicator of poor prognosis, and combined with IL-6 inhibitors or STAT3 pathway targeted drugs to guide stratified treatment (31, 32). However, for small B-cell lymphomas, enrichment of Enterobacteriaceae may serve as an early warning marker for indolent lymphoma transformation, which can delay disease progression through frequent monitoring or early intervention such as targeted antibiotics (16). The results of this study indicate that the treatment of B-cell lymphoma needs to be designed differently. For DLBCL, modulation of actinobacteria metabolism (such as propionate antagonists) or supplementation of butyrate may restore immune homeostasis; In contrast, for small B-cell lymphomas, selective elimination of enterobacteriaceae (e.g., phage therapy) may mitigate genotoxic damage (33). IL-10 reduction induced by Enterobacteriaceae may enhance the efficacy of PD-1 inhibitors, while Treg expansion mediated by Actinobacteria may limit its efficacy, suggesting that microbiota typing can optimize individualized treatment selection (34).

This study has several limitations. The relatively small sample size from a single center may limit the statistical power and generalizability of our findings, and precluded a detailed analysis of correlations with clinical parameters or investigation of rare immune cell subsets like MAIT cells. Furthermore, the 16S rRNA sequencing approach provides taxonomic profiling but not functional insights. Future multi-center studies with larger cohorts, deeper immune phenotyping, and metagenomic and metabolomic analyses are warranted to validate and extend our observations.

## 5 Conclusions

We demonstrate that untreated B-cell lymphoma patients exhibit subtype-specific gut microbial dysbiosis. Characterized by the enrichment of Selenomonadaceae and Actinobacteriota in DLBCL and Enterobacteriaceae in indolent small B-cell lymphomas, with distinct correlations to immune parameters. These microbiota signatures hold promise as biomarkers for disease stratification and prognostication. Future studies should focus on elucidating the underlying mechanisms.

## Data Availability

The original data presented in the study are openly available in the article/**Supplementary Material**.
